# Simultaneous down-regulation of tumor suppressor genes *RBSP3/CTDSPL, NPRL2/G21 *and *RASSF1A *in primary non-small cell lung cancer

**DOI:** 10.1186/1471-2407-10-75

**Published:** 2010-03-01

**Authors:** Vera N Senchenko, Ekaterina A Anedchenko, Tatiana T Kondratieva, George S Krasnov, Alexei A Dmitriev, Veronika I Zabarovska, Tatiana V Pavlova, Vladimir I Kashuba, Michael I Lerman, Eugene R Zabarovsky

**Affiliations:** 1 Laboratory of Structural and Functional Genomics, Engelhardt Institute of Molecular Biology, Russian Academy of Sciences, Moscow, Russia; 2 Microbiology and Tumor Biology Center, Department of Clinical Science and Education, Södersjukhuset, Karolinska Institute, Stockholm, Sweden; 3 Blokhin Cancer Research Center, Russian Academy of Medical Sciences, Moscow, Russia; 4 Institute of Molecular Biology and Genetics, Ukrainian Academy of Sciences, Kiev, Ukraine; 5 Cancer-Causing Genes Section, Laboratory of Immunobiology, Center for Cancer Research, National Cancer Institute, Frederick, USA

## Abstract

**Background:**

The short arm of human chromosome 3 is involved in the development of many cancers including lung cancer. Three bona fide lung cancer tumor suppressor genes namely *RBSP3 *(AP20 region),*NPRL2 *and *RASSF1A *(LUCA region) were identified in the 3p21.3 region. We have shown previously that homozygous deletions in AP20 and LUCA sub-regions often occurred in the same tumor (P < 10^-6^).

**Methods:**

We estimated the quantity of *RBSP3, NPRL2, RASSF1A, GAPDH, RPN1 *mRNA and *RBSP3 *DNA copy number in 59 primary non-small cell lung cancers, including 41 squamous cell and 18 adenocarcinomas by real-time reverse transcription-polymerase chain reaction based on TaqMan technology and relative quantification.

**Results:**

We evaluated the relationship between mRNA level and clinicopathologic characteristics in non-small cell lung cancer. A significant expression decrease (≥2) was found for all three genes early in tumor development: in 85% of cases for *RBSP3*; 73% for *NPRL2 *and 67% for *RASSF1A *(P < 0.001), more strongly pronounced in squamous cell than in adenocarcinomas. Strong suppression of both, *NPRL2 *and *RBSP3 *was seen in 100% of cases already at Stage I of squamous cell carcinomas. Deregulation of *RASSF1A *correlated with tumor progression of squamous cell (P = 0.196) and adenocarcinomas (P < 0.05). Most likely, genetic and epigenetic mechanisms might be responsible for transcriptional inactivation of *RBSP3 *in non-small cell lung cancers as promoter methylation of *RBSP3 *according to NotI microarrays data was detected in 80% of squamous cell and in 38% of adenocarcinomas. With NotI microarrays we tested how often LUCA (*NPRL2, RASSF1A*) and AP20 (*RBSP3*) regions were deleted or methylated in the same tumor sample and found that this occured in 39% of all studied samples (P < 0.05).

**Conclusion:**

Our data support the hypothesis that these TSG are involved in tumorigenesis of NSCLC. Both genetic and epigenetic mechanisms contribute to down-regulation of these three genes representing two tumor suppressor clusters in 3p21.3. Most importantly expression of *RBSP3, NPRL2 *and *RASSF1A *was simultaneously decreased in the same sample of primary NSCLC: in 39% of cases all these three genes showed reduced expression (P < 0.05).

## Background

The short arm of human chromosome 3 (region 3p21.3) contains clusters of tumor suppressor genes (TSG) involved in multiple cancer types including lung, kidney, breast, cervical, nasopharyngeal and other carcinomas [1-5]. We performed a comprehensive deletion survey of 3p in more than 400 of major epithelial cancer samples and identified two most frequently affected regions - LUCA at the centromeric and AP20 at the telomeric border of 3p21.3 [6-10]. Aberrations in these loci were detected in more than 90% of studied tumors. Homozygous deletions (HD) were frequently detected in all tumors in both the LUCA and AP20 regions. The frequent chromosome losses in these regions suggested that they harbor several multiple TSG [9,10]. More than 20 genes were localized in these two regions and among them at least three TSG were identified: *RBSP3 *(RB protein serine phosphatase from chromosome 3 gene or HYA22 or CTDSPL; CTD small phosphatase family), *NPRL2 *(nitrogen permease regulator-like 2 gene or G21 or TUSC4; NPR family) and *RASSF1A *(Ras association domain family member 1 gene). *RBSP3 *was mapped to AP20 and the others two to the LUCA region [1,11-13].

The *RBSP3 *gene occupies more than 120 kb and contains at least 8 exons coding for a 4.8 kb mRNA that is ubiquitously expressed in normal tissues including lung. By sequence analysis *RBSP3 *belongs to a gene family of small C-terminal domain phosphatases that may control the RNA polymerase II transcription machinery [14]. Two sequence splice variants of *RBSP3 *(A and B) were identified and an initial analysis of *RBSP3 *was performed in lung and other cancers [12]. The expression of the gene was greatly decreased in several small cell lung cancer (SCLC) and NSCLC cell lines. *RBSP3 *showed growth suppression with regulated transgenes in cell culture and suppression of tumor formation in SCID mice. It was demonstrated that transient expression of variant A and B resulted in drastic reduction of the phosphorylated form of RB protein presumably leading to a block of the cell cycle at the G1/S boundary. In addition, frameshift, nonsense and missense mutations in *RBSP3 *have been reported [15]. All these features are consistent with classical characteristics of a TSG.

The *NPRL2/G21 *gene covers 3.3 kb and contains 11 exons coding for the main 1.8 kb transcript with multiple splice isoforms that are expressed in all tested normal tissues including lung. By sequence analysis, the main product of *NPRL2/G21 *encodes a soluble protein that has a bipartite nuclear localization signal, a protein-binding domain, similarity to MutS core domain, and a newly identified nitrogen permease regulator 2 domain with unknown function. This information suggests that the nuclear protein NPRL2/G21 may be involved in DNA mismatch repair, cell cycle checkpoint signaling, and regulation of the apoptotic pathway. NPRL2 plays an important role in cisplatin-induced resistance in human non-small-cell lung cancer cells [16,17]. Previously obtained results indicated that *NPRL2/G21 *is a multiple tumor suppressor gene [16,18,19].

The *RASSF1 *gene occupies 7.6 kb and contains 5 exons coding for 2 kb alternatively spliced mRNAs [6,11,20]. One of the major splicing forms is *RASSF1A*. Several studies have shown that loss of *RASSF1A *expression occurs in many different cancers because of tumor acquired promoter DNA methylation and the gene is able to suppress growth of lung cancer cells in culture and tumor formation in mice [13,21-24]. For example, *RASSF1A *is silenced by promoter hypermethylation in 100% of SCLCs and in 63% of NSCLCs cell lines and in 50-100% SCLC and 21-58% NSCLC primary tumors [25-28]. As in the case of *RBSP*3, frameshift, nonsense and missense mutations in *RASSF1A *have been discovered [15,29]. The amino acid sequence of RASSF1A (340 amino acids) contains a predicted diacylglycerol (DAG) binding domain and a Ras association domain. Association of human proteins RASSF1C and RASSF1A with Ras protein was demonstrated [30,31]. RASSF1A can induce cell-cycle arrest by engaging the Rb-family cell cycle checkpoint [32]. *RASSF1A *is involved in several growth regulating and apoptotic pathways and regulates cell proliferation, cellular integrity and cell death [24,27]. These and other results strongly suggest that *RASSF1A *is an important human TSG involved in the development or progression of many epithelial tumors.

Previously only few studies were performed to compare expression of several 3p TSG in the same tumor sample [33,34]. To investigate this further we chose *RBSP3*, *NPRL2 *and *RASSF1A *and analyzed their expression by qPCR in primary tumors: non-small cell lung cancer (NSCLC) - adenocarcinoma (AC) and squamous cell lung cancer (SCC).

For the first time we found that expression of all three genes was significantly decreased in 67-85% of tested NSCLC cases. Moreover, the simultaneous down-regulation of *RBSP3*, *NPRL2 *and *RASSF1A *in the same tumor sample was observed in 39% of all cases. Both genetic and epigenetic mechanisms contributed to deregulation of these three genes representing two TSG clusters in 3p21.3.

## Methods

### Tissue specimens

Paired specimens of non-small cell lung cancer (NSCLC) tissues including 41 squamous cell carcinomas (SCC), 18 adenocarcinomas AC) and adjacent morphologically normal tissues (conventional "normal" matched control samples) were obtained after surgical resection of primary lung cancer prior radiation or chemotherapy and stored in liquid nitrogen. "Normal" matched controls were obtained minimum at 2 cm distance from the tumor and confirmed histologically as normal lung epithelial cells. The diagnosis was verified by histopathology and only samples containing 70% or more tumor cells were used in the study. The samples were collected in accordance to the guidelines issued by the Ethics Committee of Blokhin Cancer Research Center, Russian Academy of Medical Sciences (Moscow). All patients gave written informed consent that is available upon request. The study was done in accordance with the principles outlined in the Declaration of Helsinki. All tumor specimens were characterized according to the International System of Clinico-Morphological Classification of Tumors (TNM), based on the tumor-node-metastasis and staging classification of 1989 [35] and WHO criteria classification of 1999 [36]. Relevant clinical and pathological characteristics of the patients with NSCLC included in this study are summarized in Table 1. Normal lung tissues (autopsy material) were obtained post mortally from ten healthy individuals (age 23-49 lacking cancer history with absence of chronic diseases).

**Table 1:** Clinical and pathological characteristics of patients with NSCLC

Variables	Patients	
Gender/n	Female/7, Male/52	
Age	Mean 60 Range 31-76	
TNM/Stage	Histological type of NSCLC	
	SCC	AC
T_1_N_0_M_0_/Stage IA	6	5
T_2_N_0_M_0_/Stage IB	5	2
T_3_N_0_M_0_, T_2_N_1_M_0_/Stage IIB	19	5
T_1_N_2_M_0_, T_2_N_2_M_0_, T_3_N_1_M_0_, T_3_N_2_M_0_/Stage IIIA	11	5
T_4_N_0_M_0_/Stage IIIB	--	1
N_0 _Stage (no metastases)	24	10
N_1 _Stage +N_2 _Stage (with metastases)	17	8
Central cancer	27	1
Peripheral cancer	9	16
ND	5	1
Total	41	18

### DNA and total RNA extraction and reverse transcription reaction

DNA was extracted using the Dneasy Tissue kit (Qiagen, USA) and total RNA was isolated with Rneasy mini kit (Qiagen, USA) according to the manufacturer's recommendation. RNA quality was assessed with spectrophotometer NanoDrop ND-1000 (NanoDrop Technologies Inc. USA) and by gel electrophoresis. All RNA samples were treated with RNAse free DNase I (Fermentas, Lithuania) and cDNA was synthesized using MMLV reverse transcriptase and random hexamers according to standard manufacturer's protocol (Fermentas, Lithuania).

### Analysis of mRNA and DNA copy number by qPCR

The sequences of primers and probes are shown in Table 2. All reactions were performed using ABI 7000 PRISM™ SDS (Applied Biosystems) with RQ software (PCR program: 10 min at 95°C, then 40 two-step cycles 15 s at 95°C and 60 s at 60°C) in total volume 25 μl in triplicate. All probes contained the dye FAM at 5'-end and RTQ1 at 3'-end. Final concentrations of primers and probes for target and reference genes were: *RBSP3 *cDNA primers- 350 nM, probe - 150 nM; *RBSP3 *DNA primers - 150 nM, probe - 100 nM; *ACTB *DNA primers- 200 nM, probe - 100 nM, *NPRL2 *cDNA primers - 500 nM, probe 300 nM; *RASSF1A *cDNA primers - 300 nM, probe 300 nM;*GAPDH *cDNA primers - 300 nM, probe - 150 nM; *RPN1 *cDNA primers - 350 nM, probe - 200 nM;*RPN1 *DNA primers - 200 nM, probe - 100 nM; *GUSB *cDNA primers - 350 nM, probe - 250 nM; *GUSB *DNA primers - 200 nM, probe - 200 nM. PCR products were analyzed in 1.8% agarose gels and nucleotide sequences of the amplicons were verified by sequencing with 3730 DNA Analyzer automated sequencer (Applied Biosystems).

**Table 2:** Primers and probes for target and reference genes for expression levels and copy number studies

Gene	Name	Primers (F, R) and probe (Z) sequences 5' → 3'	Amplicon lengh
Target genes			
*RBSP3/CTDSPL* NM_001008392	*RBSP3 *(RB1 serine phosphatase from human chromosome 3), CTD (carboxy-terminal domain, RNA polymerase II, polypeptide A) small phosphatase-like	cDNA F: GCGAGAAAGCCTCCCAGTG R: CCACCATTCTCCTCCACCAGT Z: CCACATTGTAATCACGGAAGCAGCAGA	154
		DNA F: CAGAGTGCGTGTGCCGACT R: ACAACTTCTCTGCGGGCGT Z: CTGGCGGAGAGACTGGGAGCGA	126
*NPRL2/G21* NM_006545	Nitrogen permease regulator-like 2 gene	cDNA F: GGACCTCACTACACAACAAATCCTG R: GTCACAACGCCGTAGTACAGCA Z: ACATCCAGAAGATTTCAGCAGAGGCAGAT	134
*RASSF1A* NM_007182	Ras association (RalGDS/AF-6) domain family 1	cDNA F: CGCGCATTGCAAGTTCAC R: AGGCTCGTCCACGTTCGT Z: CGCTCGTCTGCCTGGACTGTTGC	120
Reference genes			
*RPN1* NM_002950	Ribophorin I	cDNA F: CACCCTCAACAGTGGCAAGAAG R: TGCATTTCGCTCACTCTGTCG Z: CCCTCTGTCTTCAGCCTGGACTGC	125
		DNA F: TATGGGCCTTTCAGAGATGTGCCT R: ACCACCCAAGCCTATCAACCAGTA Z: TGGAGTCCAGCCCATCCCTGTCTGCTTCA	120
*GUSB* NM_000181	Glucuronidase, beta β-D-glucuronidase	cDNA F: GATGGAAGAAGTGGTGCGTAGG R: TTAGAGTTGCTCACAAAGGTCACAG Z: CGTCCCACCTAGAATCTGCTGGCTACTACTT	171
		DNA F: TGCCGTGAGTCTCTGCTGTG R: CCTACGCACCACTTCTTCCATC Z: TGACCCTCTGTCCCTTCCCTCCTG	151
*ACTB* NM_001101	Actin, ß	DNA F: GTGCTCAGGGCTTCTTGTCCTTT R: TTTCTCCATGTCGTCCCAGTTGGT Z: AAGGATTCCTATGTGGGCGACGAGGCCCA	160
*GAPDH* NM_002046	Glyceraldehyde-3-phosphate dehydrogenase	cDNA F: GGAGTCAACGGATTTGGTC R: TGGGTGGAATCATATTGGAACAT Z: CCTTCATTGACCTCAACTACATGGTTTACAT	139

qPCR data were analyzed using the relative quantification or ΔΔC_t_-method [37,38] based on mRNA (or DNA) copy number ratio (R) of a target gene versus reference gene in a given tumor sample relative to matched normal control sample (see above Tissue specimens section) according to the formula:

where *E *- efficiency of reaction, *C*_*T *_- threshold cycle, ref - reference gene, tar - target gene.

All preliminary validation steps have been done: standardization of all assays, reproducibility of the qPCRs in parallel and in independent runs, selection of reference samples and testing of reference genes http://www.gene-quantification.info/.

### NotI-microarray analysis

Microarrays were constructed essentially as previously described [39,40]. In brief, two oligonucleotides:

NotX: 5'-AAAAGAATGTCAGTGTGTCACGTATGGACGAATTCGC-3'

and NotY: 5'-GGCCGCGAATTCGTCGGTATGCACTGTGTGTGACATTCAAA-3"

were used to create the NotI linker. Annealing was carried out in a final volume of 100 μl containing 20 μl of 100 μM NotX, 20 μl of 100 μM NotY, 10 μl of 10×M buffer (Roche Molecular Biochemicals) and 50 μl of H_2_O. Two micrograms of tumor and normal control DNA (50 μg/ml) were digested with 20 U of *Sau*3A (Roche Molecular Biochemicals) at 37°C for 5 h and then 0.4 μg of the digested DNAs were circularized overnight with the T4 DNA ligase (Roche Molecular Biochemicals) in the appropriate buffer in 1 ml reaction mixture. Then DNA was concentrated with ethanol, partially filled in and digested with 10 U of *Not*I at 37°C for 3 h. Following digestion, *Not*I was heat inactivated and DNAs were ligated overnight in the presence of a 50 M excess of NotI linker at room temperature. NotI- representation (NR) probes were labeled in a PCR reaction with *Not*X primer. The majority of products of the DNA amplification step were in the 0.2-1.0 kb range. Repeated PCR was conducted for labeling NR with fluorophores.

Hybridization of coupled normal/tumor NotI samples was carried out at 42°C for 15 h in a Lucidea Base device (Amersham Pharmacia Biotech) according to manufacturer's recommendations. Automatic washing of the microarrays was performed in the same device using manufacturer's protocol. The following solutions were sequentially used for the washing: 1) 0.2% SDS+1 SSC; 2) 0.2% SDS+0.1 SSC; 3) 0.1 SSC; 4) de-ionized water; 5) isopropyl alcohol. Then microarrays were scanned in the GenePix 4000 A and results were processed with GenePix Pro 6.0 software (Amersham Pharmacia Biotech).

### Statistical analysis

Nonparametric Wilcoxon test was used to compare mRNA expression differences of target and reference genes for the same NSCLC sample. Then groups of samples were compared in respect to average level of mRNA decrease (LD_av_) and the frequency of decrease (FD). The LD was calculated as 1/R and reflects the n-fold factor by which the mRNA content decreased in the tumor compared to normal tissue. Nonparametric Kruskal-Wallis and Mann-Whitney rank-sum tests were used to test mRNA differences (both LD_av _and FD) for each target gene in NSCLC (AC, SCC) and with and without metastases. Nonparametric Spearmen's criterion was used to calculate the coefficient of correlation between the levels of mRNA decrease (LD_av_) for each set of pairs of target genes. P-values < 0.05 were considered statistically significant. All statistical procedures were performed using the BioStat software [41].

## Results

### Expression of RBSP3, NPRL2 and RASSF1A genes in lung tissues of healthy donors

**Figure 1:**
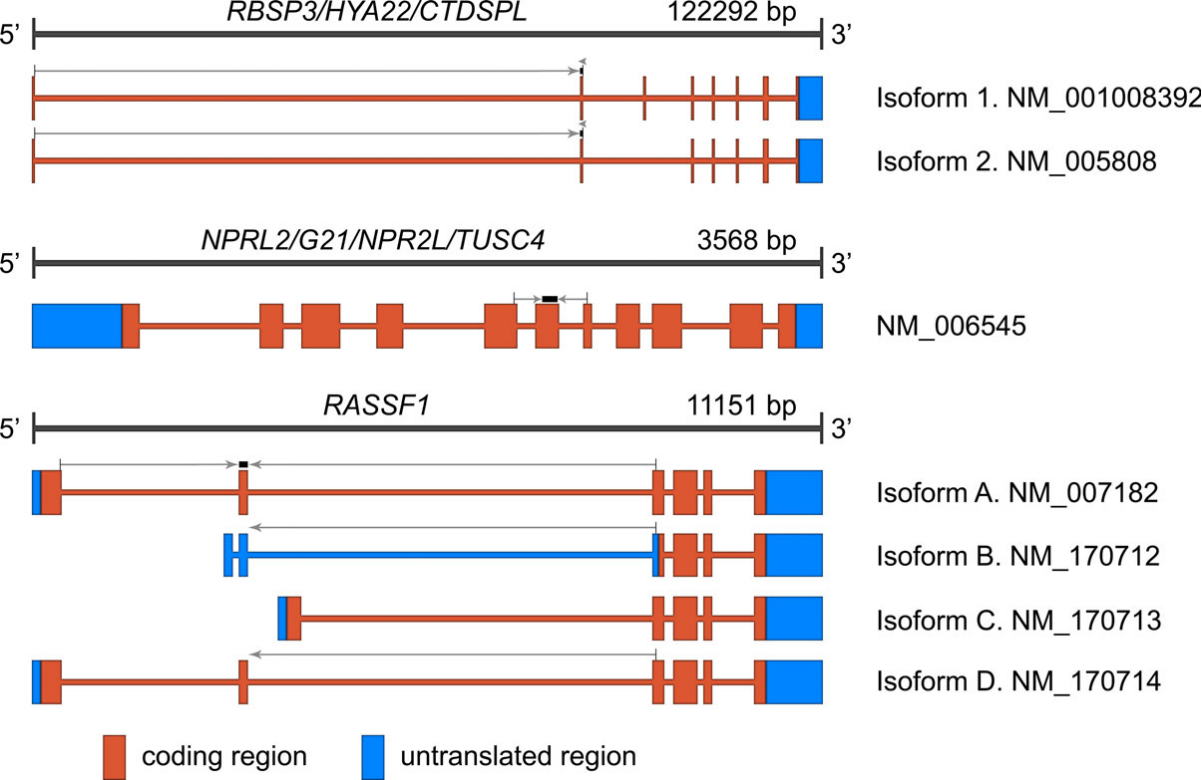
**Schematic representation of the *RBSP3 *(A), *NPRL2*/*G21*/*NPR2L*/*TUSC4 *(B), and *RASSF1A *(C) genes**. Exon-intron structure of known mRNAs is shown. Coding and non-coding mRNA regions are depicted by dark and light grey boxes, respectively. Primers and probes designed for qPCR are shown by arrows and boxes above mRNAs. A. *RBSP3 *(3p21.3, AP20 region). Forward primer crossed the 1^st^/2^nd ^exons boundary, reverse primer and probe were located in the 2^nd ^exon. B. *NPRL2 *(3p21.3, LUCA region): Forward primer crossed the 5^th^/6^th ^exons boundary, reverse primer crossed the 6^th^/7^th ^exons boundary, probe was located in the 6^th ^exon. C. *RASSF1A *(3p21.3, LUCA region): Forward primer crossed the 1^st^/2^nd ^exons boundary that provides specificity to isoforms A and E. Reverse primer crossed the 2^nd^/3^rd ^exons boundary that provides specificity to isoforms A, B and D. Combination of forward and reverse primers provided specificity to isoform A only. Probe was located in the 2^nd ^exon.

Genomic structure and location of primers and probes is shown in Figure 1.

It is known that normally looking cells surrounding tumors can already harbor genetic and epigenetic changes compared to the cells isolated from the same tissue from healthy individuals. Therefore we decided to check expression of these 3 genes in samples obtained from healthy donors and in biopsies of "normal" (matched) control samples obtained from patients with NSCLC. The mRNA levels were measured in lung tissues from healthy donors using different reference samples. RNA pool isolated from lung tissues of 10 healthy donors was used in one set of experiments and in another test the same samples were examined against RNA pool isolated from adjacent to tumor "normal" tissues of 30 NSCLC patients. The *GAPDH *was used as a reference gene and its expression did not vary more than 2-fold in all samples examined. Very similar results were obtained for target genes in both experiments. We concluded that expression of all three genes did not significantly vary (R = 1.5 ± 0.5) in normal lung tissues from healthy donors and patients with NSCLC ("normal" matched controls).

### Expression of three TSGs in primary NSCLC - AC and SCC

**Figure 2:**
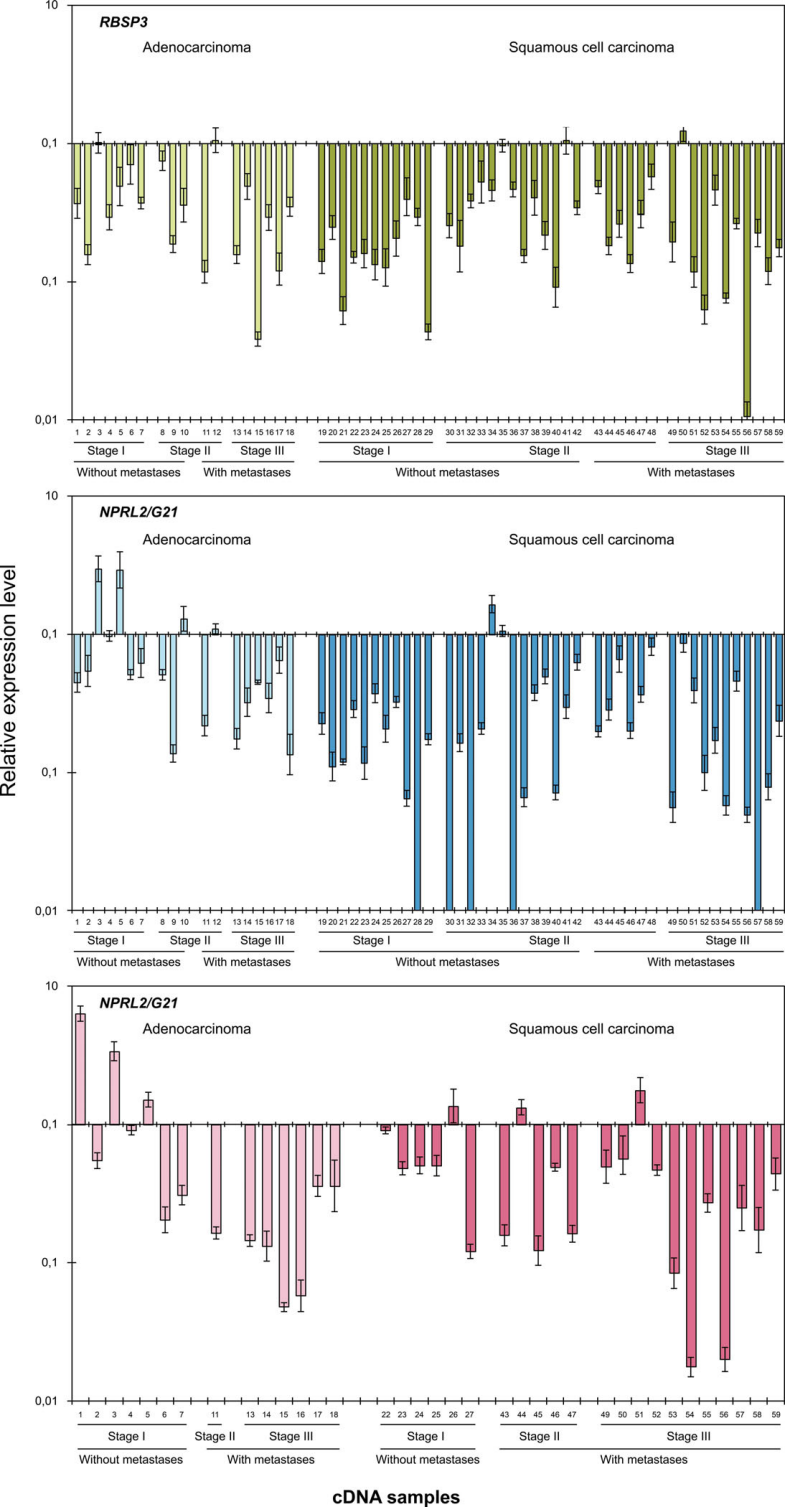
**The relative expression levels of *RBSP3*, *NPRL2*/*G21 *and *RASSF1A *genes in AC and SCC**. The mRNA level of each target gene was normalized by that of the reference gene *GAPDH*. Data for ADC and SCC were presented according to cancer progression stages and to the presence of metastases.

**Figure 3:**
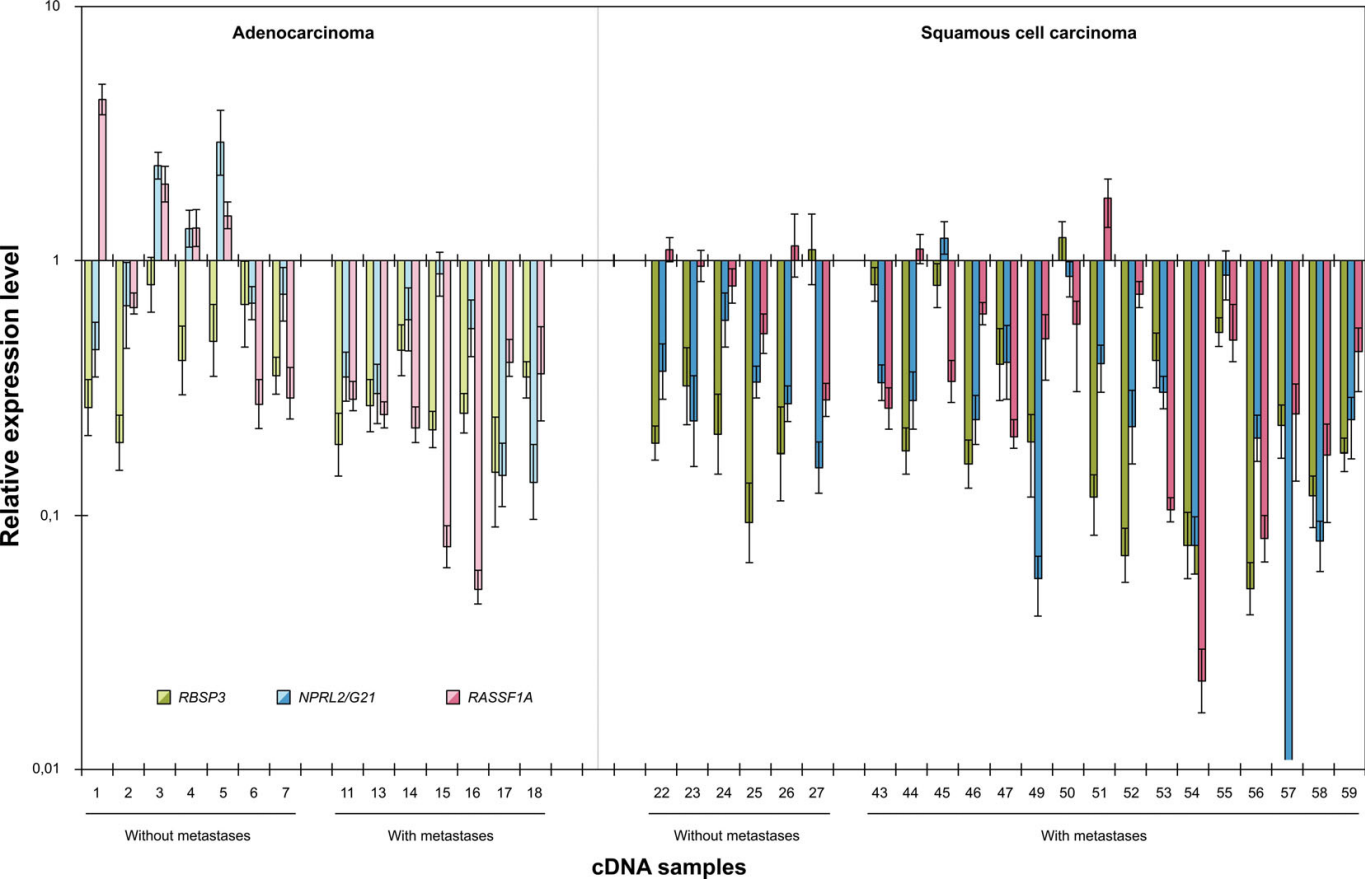
**Simultaneous down-regulation of tumor suppressor genes *RBSP3/CTDSPL*, *NPRL2/G2,1 *and *RASSF1A *in primary non-small cell lung cancer (AC and SCC)**. The data were for the same 36 primary tumor samples as the data presented in Figure 2. The mRNA level of each target gene was normalized by those of two reference genes *GAPDH *and *RPN1*.

The results of mRNA level quantification for three genes and statistical analysis are shown in Figures 2 and 3 and Table 3. The variability of *GAPDH *and *RPN1 *mRNA was less than 2-fold in studied samples, therefore in this study we considered significant greater than or equal to 2-fold decrease of target genes expression.

**Table 3:** The frequency of mRNA decrease (FD) and the average level of mRNA decrease (LD_av_)

Clinical stage/Groups		**Frequency (FD)**^**a **^**and level of mRNA decrease (LD**_**av**_**)**^**b**^					
		*RBSP3* (59 tumors)		*NPRL2* (59 tumors)		*RASSF1A* (36 tumors)	
		AC	SCC	AC	SCC	AC	SCC
I	FD LD_av_	*71 (5/7) *3 (2 -- 6)	*100 (11/11) *7 (2 -- 23)	14 (1/7) 2	*100 (11/11) *7 (2 -- 100)	29 (2/7) 4 (3 -- 5)	33 (2/6) 4 (2 -- 8)
II	FD LD_av_	*60 (3/5) *5 (2 -- 9)	*79 (15/19) *4 (2 -- 11)	40 (2/5) 6 (5--7)	*74 (14/19) *9 (2 -- 100)	100 (1/1) 6	80 (4/5) 5 (2 -- 8)
III	FD LD_av_	*100 (6/6) *6 (2 -- 26)	*91 (10/11) *8 (2 -- 94)	*83 (5/6) *4 (2 -- 7)	*91 (10/11) *10 (2 -- 100)	*100 (6/6) *7 (3 -- 21)	*82 (9/11) *7 (2 -- 57)
Group without metastasis	FD LD_av_	*70 (7/10) *3 (2 -- 6)	*88 (21/24) *5 (2 -- 23)	*20 (2/10) *4 (2 -- 7)	*88 (21/24) *10 (2 -- 100)	**29 (2/7)** **4 (3 -- 6)**	*33 (2/6)* *4 (2 -- 8)*
Group with metastasis	FD LD_av_	*88 (7/8) *6 (2 -- 26)	*88 (15/17) *7 (2 -- 94)	*75 (6/8) *4 (2 -- 7)	*82 (14/17) *8 (2 -- 100)	*100 (7/7) *7 (3 -- 21)	*81 (13/16) *6 (2 -- 57)
Total	FD LD_av_	*78 (14/18) *4 (2 -- 26)	*88 (36/41) *6 (2 -- 94)	*44 (8/18) *4 (2 -- 7)	*85 (35/41) *9 (2 -- 100)	*64 (9/14) *6 (3 -- 21)	*68 (15/22) *6 (2 -- 57)

^a ^-- Frequencies of expression decrease are shown as percentages. The number of cases with decreased expression and all tested samples are shown in parentheses.

^b ^-- Level of expression decrease in tumor samples is shown in n-fold relative to expression in normal controls

* -- cases when decrease of target gene expression (FD and LD_av_) in tumor samples relative to normal controls was statistically valid according to the non-parametric Wilcoxon test (P ≤ 0.05 -0.001)

Bold font designates cases when: (i) FD and LD_av _for *NPRL2 *genes was more strongly pronounced in SCC than in AC samples according to the Mann -Whitney test (P = 0.002) and (ii) the difference of the FD and LD_av _for *RASSF1A *gene was statistically valid during AC progression according to the Mann -Whitney test (P < 0.05)

Underlined italics highlights positive tendency of FD and LD_av _differences of *RBSP3, NPRL2 *and *RASSF1A *genes during progression (AC and SCC respectively)

#### RBSP3

Significant (from 2 to 94-fold) decrease of the mRNA level (LD) was observed in 85% (50 of 59, P < 0.001) of all NSCLC cases. Frequency of mRNA decrease (FD) was high in both AC (78%) and SCC (88%). Interestingly at Stage I of the SCC cases expression of *RBSP3 *was down-regulated already in 100% cases and only in 71% of AC samples (Table 3). On the other hand the tendency of more severe deregulation of *RBSP3 *transcription during tumor progression was more evident for AC cases: in average a 3-fold declined expression in 70% of cases without metastases and a 6-fold in 88% of samples with metastases. The same was correct for cases at Stage I (LD_av _= 3, FD = 71%) and Stage III (LD_av _= 6, FD = 100%). Spearmen's coefficient of reverse correlation (r_s_) between tumor Stages (I, II, III) and expression R values was 0.36 (P = 0.2, i.e. non-significant).

#### NPRL2

Significant decrease of LD (from 2 to 100-fold) was seen in 73% (43 of 59, P < 0.001) of all NSCLC cases. Down-regulation of *NPRL2 *was more significant in SCC compared to AC: 9-fold and 85% vs. 4-fold and 44%, respectively, P = 0.002 (see Table 3). No expression was found in 12% (5 of 59) of SCC cases. The FD was comparable in SCC cases with metastases and without (82% and 88%, respectively). However FD in AC samples without metastases was less than with metastases (20% against 75%; P = 0.08). Again at Stage I of the SCC cases expression of *NPRL2 *was down-regulated already in 100% cases and in AC only 14% samples showed decreased expression. The tendency of more frequent decrease of *NPRL2 *mRNA level with tumor progression was seen only for AC cases, where Spearmen's coefficient of reverse correlation (r_s_) between tumor Stages (I, II, III) and expression R values was 0.47 (P = 0.06). Eighty three percent of AC cases had decreased expression at Stage III as compared to 14% at Stage I.

#### RASSF1A

Significant decrease of LD (from 2-fold to 57-fold) was detected in 67% (24 of 36, P < 0.01) of all NSCLC cases, including 64% of AC and 68% of SCC samples (Figure 2, Table 3). More severe deregulation of *RASSF1A *expression was observed during tumor progression both in AC and SCC. Spearmen's coefficient of reverse correlation (r_s_) between tumor Stages (I, II, III) and R values was 0.54 (P = 0.05) for AC. Statistically significant decrease of LD_av _and increase of FD was observed in AC for patients with metastases and without (7-fold and 100% vs. 4-fold and 29%, P < 0.05). The same tendency was seen for LD_av _and FD in SCC cases with and without metastases: namely, 6-fold and 81% vs. 4-fold and 33%, respectively; P = 0.196. Moreover an average decrease of *RASSF1A *mRNA (LD_av_) in SCC at Stage I was 4-fold and at Stage III it was already 7-fold.

FD increased for both SCC (33% vs. 82%) and AC (29% vs. 100%) during tumor progression from Stage I to Stage III.

Surprisingly a 3-to-4 fold increase of mRNA level of two genes - *NPRL2 *and *RASSF1A *was found for several AC samples at Stage I (Figure 2, samples 1, 3, 5). These tumor samples were highly differentiated in contrast to samples 2, 4, 9, 10 and others that displayed low degree of differentiation and significant decrease of *NPRL2 *and *RASSF1A*. Some of them showed so high degree of anaplasia that the epithelial phenotype was almost undetectable. These undifferentiated cells were highly malignant and showed the strongest decrease of *NPRL2 *and *RASSF1A *expression.

### Simultaneous down-regulated expression of three genes in the same tumor samples

As expression of all three target genes was frequently decreased in the same NSCLC samples we tested the probability that this decrease was not random. To do this we used 36 NSCLC samples and two control reference genes *GAPDH *and *RPN1 *(Figure 3, Table 4). Results demonstrated that this simultaneous down-regulation was very frequent and statistically valid: all three genes had reduced expression (≥2) in 39% (P < 0.001) of NSCLC specimens (36% in AC and 41% in SCC). Simultaneous decreased expression of *RBSP3 *and *NPRL2 *was observed in 61% of cases and for *RBSP3 *and *RASSF1A *this occurred in 50%. In the case of *NPRL2 *and *RASSF1A *simultaneous down-regulation was detected in 44% of tumors (Table 4). Spearmen's coefficient values r_s _for *RBSP3, NPRL2/G21, RASSF1A *gene pairs were high (0.63 - 1.0, P < 0.001) in different groups especially in AC and SCC with metastases.

**Table 4:** The frequencies of simultaneous mRNA level decreases (FD) for the combination of genes *RBSP3, NPRL2/G21 *and *RASSF1A *in different groups

Groups		^**a**^**FD, (%)**			
		*RBSP3* */NPRL2/G21*	*RBSP3/* *RASSF1A*	*RASSF1A/* *NPRL2/G21*	*RBSP3/NPRL2/G21* */RASSF1A*
AC	I	14 (1/7)	14 (1/7)	0 (0/7)	0 (0/7)
	II+III	**71 **(5/7) **r**_s_**= 0.88**	**100 **(7/7) **r**_s_**= 0.88**	**71 **(5/7) **r**_s_**= 0.74**	71 (5/7)
	Total	43 (6/14)	57 (8/14)	36 (5/14)	36 (5/14)
					
SCC	I	67 (4/6)	17 (1/6)	33 (2/6)	17 (1/6)
	II+III	**75 **(12/16) **r**_s_**= 1.00**	**56 **(9/16) **r**_s_**= 0.88**	**56 **(9/16) **r**_s_**= 0.88**	50 (8/16)
	Total	**73 **(16/22) **r**_s_**= 0.94**	45 (10/22) r_s _= 0.74	50 (11/22) r_s _= 0.69	41 (9/22)
					
NSCLC	I	38 (5/13)	15 (2/3)	15 (2/3)	8 (1/13)
	II+III	74 (17/23)	70 (16/23)	61 (14/23)	57 (13/23)
	Total	61 (22/36) r_s _= 0.72	50 (18/36) r_s _= 0.68	44 (16/36) r_s _= 0.63	39 (14/36)

^a^Number of cases with reduced mRNA level/to all cases is shown in parenthesis

^b^Group I without metastases, group II+III -- with metastases

^c^The r_s _are the Spearmen's coefficient values of rank correlation. Bold designates the highest correlations between pairs of genes. P < 0.001 in all groups

### Decreased expression of RBSP3, NPRL2 and RASSF1A in primary NSCLC can be caused by genetic and epigenetic factors

To understand the mechanism underlying the observed down-regulation of *RBSP3 *in NSCLC samples we used qPCR to test DNA copy number changes, i.e. genetic factors [9,10] and NotI microarrays to examine methylation i.e. epigenetic factors [39]. Methylation was detected in 38% of AC (3 of 8) and in 80% of SCC (8 of 10) cases with decreased expression of *RBSP3*. Deletions were detected in 25% of AC samples and in 30% of SCC tumors (Table 5). Both methylation and hemizygous deletions were observed in two SCC specimens. Methylation or/and deletions of *RBSP3 *were detected in 63% of AC and in 90% of SCC cases. The data suggested that both genetic and epigenetic mechanisms are important for transcriptional inactivation of *RBSP3 *in NSCLC.

**Table 5:** Comparison of qPCR data and NotI-microarrays for *RBSP3 *gene

Sample number	qPCR		^**b**^**Not I-microarrays**	Possible reason of mRNA decrease
	mRNA decrease	^**a**^**DNA** copy number		
AC				
1	0.39	+	+	deletion
2	0.15	-	+	methylation
4	0.29	-	-	another mechanism
5	0.49	+	+	deletion
7	0.37	-	-	another mechanism
11	0.12	-	-	another mechanism
16	0.29	-	+	methylation
17	0.12	-	+	methylation
SCC				
23	0.16	-	+	methylation
25	0.13	+	+	deletion and methylation
26	0.21	-	+	methylation
27	0.39	+	+	deletion and methylation
43	0.48	-	+	deletion
46	0.14	-	+	methylation
52	0.06	-	+	methylation
53	0.46	-	+	methylation
54	0.08	-	+	methylation
55	0.26	-	-	another mechanism

^a ^+ deletion, - retention

^b^+ deletion and/or methylation, - no changes

Using NotI microarrays we cannot test methylation of *NPRL2 *and *RASSF1A *promoters because they don't contain NotI sites. However we can check genes *SEMA3F *and *GNAI2 *that are located in the same LUCA sub-region as *NPRL2 *and *RASSF1A*. We tested how often deletions and methylations occurred in the same NSCLC sample at both LUCA and AP20 sub-regions and found that this occurred in 58% of all studied cases (15 of 26). Thus, most likely both genetic and epigenetic mechanisms are responsible for simultaneous down-regulation of expression of these three genes.

## Discussion

Several candidate TSG from the 3p21.3 AP20 and LUCA sub-regions were examined in the gene inactivation test, GIT [2,12,13,16,34,42,43]. The test is based on the functional inactivation of analyzed genes that can be achieved in different ways: by mutation, deletion, methylation etc. According to these results, at least three genes can now be considered as bona fide lung TSG: NPRL2 and RASSF1A from LUCA and RBSP3 from AP20 sub-regions [12,13,16].

Earlier the decrease of RBSP3 expression was shown in SCLC, NSCLC, cervical, renal, breast, ovary, leukemia cell lines and primary tumors by Northern blot analysis, RT-PCR and qPCR [12,44-46]. The decrease or absence of *NPRL2/G21 *expression was detected in some SCLC, NSCLC and renal cancer cell lines using Northern blot analysis [6,16]. The decrease or absence of *RASSF1A *expression was found in SCLC, NSCLC and many other tumors and cancer cell lines [see [24,27,28]].

It was reported that promoter methylation was the main mechanism of *RASSF1A *loss of expression in lung cancer (see Introduction). Homozygous deletion of 3'-part of *NPRL2 *gene and rare mutations were found in NSCLC and SCLC cell lines [6,16]. There are no methylation data explaining the loss of *RBSP3 *expression in lung cancer. However frequent deletions and mutations were reported [10,12,15]. In some leukemia cell lines (up to 98%) and acute leukemia lymphoma blood samples (24%) methylation of the promoter region of *RBSP3 *was reported [44]. Methylation (up to 26%), deletions and decreased expression of *RBSP3 *were significantly associated with poor prognosis of cervical cancer [45]. Thus, inactivation of *RBSP3 *might be one of the early events in cervical carcinogenesis.

Loss of heterozygosity and quantitative real-time PCR demonstrated that aberrations in both LUCA and AP20 sub-regions occurred simultaneously in the same tumor with high probability. Thus, it was suggested that aberrations in both LUCA and AP20 sub-regions could be linked [9,10]. Indeed, homozygous deletions in both regions often occur in the same tumor (P < 3 × 10^-7^). The estimation of possible interdependency between all aberrations in the loci NLJ-003 (AP20) and NL3-001 (LUCA) as different events was carried out using a permutation test for four types of cancers: lung, renal, breast and ovarian. This test also revealed a significant correlation between different aberrations in these two loci (P < 10^-6^). The same results were obtained using Pearson correlation for numeric values of copy number changes of these loci. Indeed, proteins *RBSP3 *and *RASSF1A *could collaborate in cell cycle arrest: *RASSF1A *by inhibiting cyclin D1 [32] and *RBSP3 *by dephosphorylating pRB [12]. Thus functional collaboration of these two genes could result in activation of the RB1 gene.

In this study we tested the hypothesis that TSG in AP20 and LUCA regions were not only deleted but their expression could also be simultaneously down-regulated in NSCLC. This suggestion was indirectly supported by other studies that showed that genes over large chromosomal regions could be regulated in a coordinated fashion [33,47-49].

First we found that expression of all three genes is rather uniform in lung samples isolated from healthy donors and from normally looking lung samples obtained from NSCLC patients ("normal" matched control samples). Thus adjacent morphologically normal tissues from the patients can be used as paired reference controls to tumor samples. In the study two parameters were analyzed - the level of mRNA decrease and frequency of mRNA decrease in two major NSCLC histological subtypes (AC and SCC) and their subgroups with different characteristics such as clinical Stage, grade, tumor localization, presence of metastases and others. Although both parameters reflect deregulation of gene expression, they are not randomly but rather functionally related.

Expression analysis of the three genes revealed the following main features.

1. Expression of the three studied TSG was significantly decreased in NSCLC: 85% for *RBSP3*, 67% for *RASSF1A *and 73% for *NPRL2 *(P < 0.001). It was statistically valid both for SCC and AC.

2. Down-regulation of the three genes was already evident at Stage I of NSCLC samples. Statistically significant down-regulation of both *NPRL2 *and *RBSP3 *was seen in 100% cases at Stage I of SCC.

3. The degree and frequency of the expression decrease for all three genes was more strongly pronounced in SCC than in AC samples (see Table 3). This difference was statistically valid in the case of *NPRL2 *(P = 0.002).

4. All studied genes were involved in progression of AC. The tendency of more severe expression down-regulation of the *RBSP3 *was evident during tumor progression of AC with respect to FD and LD (70% and 3-fold in cases without metastases in contrast to 88% and 6-fold decrease in cases with metastases, P = 0.13). For *NPRL2*, this tendency was also seen only for AC - 83% of cases had decreased expression at Stage III compared to 14% at Stage I and in 75% of AC cases with metastases vs. 20% of cases with metastases, P = 0.08, see Table 3). Expression of *RASSF1A *revealed the most strongly pronounced correlation between decrease of expression (FD and LD) and tumor progression both in SCC and AC. For example, difference in FD values was obvious between cases with and without metastases. For SCC cases this difference was 33% vs. 81% (P = 0.196) and for AC it was even more sharp, 29% vs. 100% (P < 0.05).

5. Expression of *RBSP3 *and *RASSF1A *was most seriously affected in respect to FD. For *RBSPS3 *it was detected in 85% of all NSCLC cases and for *RASSF1A *in 67%. However, regarding LD, the expression of *NPRL2 *was most strongly inhibited (LD_av _= 8), while *RBSP3 *showed weaker inhibition (LD_av _= 5).

6. Preliminary data suggested that no statistically significant difference was observed in cases with relation to age, smoking history and other cytological and pathological characteristics.

7. NotI microarrays and qPCR on genomic DNA we tested for possible mechanisms of the declined expression of *RBSP3 *in NSCLC. The data suggested that both genetic and epigenetic mechanisms were important for transcriptional inactivation of *RBSP3 *in NSCLC. Altogether deletions were detected in 25% of AC samples and in 30% of SCC patients. Methylation of *RBSP3 *was detected in 38% of AC and in 80% of SCC cases. With NotI microarrays we also tested how often LUCA (*NPRL2 *and *RASSF1A*) and AP20 (*RBSP3*) regions were deleted or methylated in the same tumor and found that this occurred in 58% of all studied cases (18 of 26). Thus, most likely both genetic and epigenetic mechanisms are responsible for simultaneous down-regulation of expression of these three TSG.

## Conclusion

The detailed analysis of mRNA expression levels of three 3p TSG was performed in two histological subtypes of NSCLC - AC and SCC respectively. The most important finding was that expression of *RBSP3, NPRL2 *and *RASSF1A *decreased in the same samples of primary NSCLC: all 3 genes have reduced expression in 39% of cases (P < 0.05). Declined expression of *RBSP3 *and *NPRL2 *was observed in 61% of samples (P < 0.05), *RBSP3 *and *RASSF1A *in 50% (P < 0.05), and *NPRL2 *and *RASSF1A *in 44% (P < 0.05) of cases. *RASSF1A *and *NPRL2 *are located in the same locus and if close localization is the reason for simultaneous decrease of expression then such decrease should be significantly less frequent when genes from the LUCA region are compared with *RBSP3*. However this was not the case and thus close localization is not the main factor for simultaneous decrease of expression. At present expression of many thousands of genes in NSCLC is investigated using cDNA microarrays [50,51]. These experiments already produced very valuable data. However, results of different studies varied significantly. They should be proved by independent methods and qPCR is one of the methods of choice that allows more detailed analysis of particular genes. This work is one of such studies. All presented data confirm and extend our previous results for lung, renal, breast, cervical cancers [9,10] and support the hypothesis that two TSG clusters (in AP20 and LUCA) are very likely co-regulated by common mechanisms.

## Abbreviations

qPCR: quantitative real-time reverse transcription-polymerase chain reaction; NSCLC: non-small cell lung cancer; AC: adenocarcinoma; SCC: squamous cell carcinoma; RBSP3: RB1 serine phosphatase from human chromosome 3; NPRL2: nitrogen permease regulator-like 2 gene; RASSF1A: Ras association domain family 1A; ACTB: beta actin; GAPDH: Glyceraldehyde-3-phosphate dehydrogenase; RPN1: Ribophorin 1; GUSB: Glucuronidase, beta.

## Competing interests

The authors declare that they have no competing interests.

## Authors' contributions

VNS designed the experiments, carried out the qPCR analysis, and wrote the manuscript; EAA collected and classified all lung tumors, performed the qPCR data; TTK - contributed with clinical information for these patients and drafting the manuscript; GSK - was responsible for primers and probes design and analyzed the data; AAD -was responsible for calculations and statistical analysis. VIZ, TVP, VIK performed the experiments and analyzed the NotI-data; MIL helped in the evaluation of the results and revised the manuscript critically for important intellectual content; ERZ was responsible for design study and coordination, wrote the manuscript. All authors read and approved the final manuscript.

## Pre-publication history

The pre-publication history for this paper can be accessed here:

http://www.biomedcentral.com/1471-2407/10/75/prepub
